# Characterization of Clinical Manifestations in the Co-occurring Phenotype of Attention Deficit/Hyperactivity Disorder and Autism Spectrum Disorder

**DOI:** 10.3389/fpsyg.2020.00861

**Published:** 2020-05-15

**Authors:** Alessandra Carta, Elisa Fucà, Silvia Guerrera, Eleonora Napoli, Giovanni Valeri, Stefano Vicari

**Affiliations:** ^1^Department of Neurosciences, Child and Adolescent Neuropsychiatry Unit, Bambino Gesù Children’s Hospital, IRCCS, Rome, Italy; ^2^Section of Child Neuropsychiatry, Department of Medical, Surgical and Experimental Sciences, University of Sassari, Sassari, Italy; ^3^Department of Life Sciences and Public Health, Catholic University of the Sacred Heart, Rome, Italy

**Keywords:** neurodevelopmental disorders, psychopathological profile, behavioral problems, autism spectrum disorder, attention deficit hyperactivity/impulsivity disorder, externalizing problems, internalizing problems

## Abstract

Comorbidity between attention deficit/hyperactivity disorder (ADHD) and autism spectrum disorder (ASD) is a frequently reported condition. However, the clinical overlaps between the two disorders are not well characterized. The Child Behavior Checklist (CBCL) is a well-documented measure of emotional and behavioral problems in children and adolescents. The aim of the present study was to evaluate whether CBCL scales were able to detect psychopathological comorbidities as well as emotional and behavioral profiles across three groups of children with ASD, ADHD, and with the co-occurrence of both disorders. The results show that around 30% of participants with ASD exhibited internalizing problems, which was in line with previous findings. Co-occurrence condition showed a clinical intermediate phenotype: relative to ADHD and ASD, youths with co-occurrence of ADHD and ASD phenotype showed respectively lower (*p* < 0.000) and higher externalizing problems (*p* < 0.000). No differences emerged in internalizing problems (*p* > 0.05) across groups. CBCL is a useful measure to study the psychopathological conditions as well as emotional and behavioral profiles associated with ASD, ADHD, and the co-occurrence of ADHD and ASD. The identification of psychopathological and behavioral profiles associated with ASD and ADHD is crucial to perform specific and individualized treatments. Our preliminary findings suggested the existence of an intermediate and independent phenotype between ADHD and ASD that seems to be defined by the externalizing problems. Internalizing problems do not significantly differ between the combined phenotype and the two groups.

## Introduction

Attention deficit/hyperactivity disorder (ADHD) and autism spectrum disorder (ASD) are common neurodevelopmental disorders (American Psychiatric Association [APA], 2013) that frequently co-occur (Lai et al., 2019).

Attention deficit/hyperactivity disorder is a prevalent and persistent psychiatric disorder that emerges in childhood as a complex of symptoms characterized by developmentally inappropriate and impairing levels of hyperactivity, impulsivity, and/or inattention (American Psychiatric Association [APA], 2013). Polanczyk et al. (2015) estimated that the worldwide prevalence in childhood population studies is around 5%. This result is in line with all previously reported systematic reviews, which estimated the prevalence of ADHD in the pediatric population as 3.4% (95% CI 2.6–4.5), with heterogeneity in methods between studies cited as a reason for different prevalences shown (Polanczyk et al., 2015).

Autism spectrum disorder is characterized by persistent deficits in social communication and social interaction across multiple contexts as well as restricted, repetitive patterns of behavior, interests, or activities (American Psychiatric Association [APA], 2013). The last reported prevalence based on The Autism and Developmental Disabilities Monitoring (ADDM) Network (Christensen et al., 2018) estimated that ASD prevalence is around 1:59 children. European studies vary between 1 and 2% of the childhood population. Differences in prevalence estimates vary by methodological approach, demographic factors, geographical area, and time (Lyall et al., 2017).

Both conditions are characterized by a high rate of psychiatric comorbidities. These affect around 80% of youth with ADHD (Banaschewski et al., 2011), and approximately two-thirds of patients with ASD are indeed reported to have at least one associated mental health condition (Simonoff et al., 2008; Lai et al., 2014). ADHD and ASD show an overlap in symptoms, such as inattention and hyperactivity/impulsivity (Taylor et al., 2015; Ghirardi et al., 2019), and ADHD has been found to be the most frequently diagnosed disorder in co-occurrence with ASD (Lai et al., 2019).

Despite significant overlap in symptoms, the previous diagnostic criteria [Diagnostic and Statistical Manual of Mental Disorders Fourth Edition, Text Revision (DSM-IV-TR); American Psychiatric Association [APA], 1994] prohibited the simultaneous diagnosis of both disorders. In the context of revised criteria in the Diagnostic Statistical Manual of Mental Disorders Fifth Edition (DSM-5), a combined diagnosis is allowed (American Psychiatric Association [APA], 2013). Consequently, a growing interest in an in-depth characterization of the co-occurring phenotype has been observed. Indeed, co-occurred diagnosis of ADHD and ASD has been frequently described in several previous studies. Autistic symptoms co-occurred in 20–63% of children with ADHD (Ronald et al., 2008; Simonoff et al., 2008; Banaschewski et al., 2011; Lecavalier et al., 2019) and attention deficit and hyperactivity-impulsivity symptoms in a range between 22 and 83% of children among those with ASD (Ronald et al., 2008; Simonoff et al., 2008; Lai et al., 2014). Symptoms of ASD may frequently be misdiagnosed with a sole diagnosis of ADHD and vice versa in young children (Matson et al., 2013).

In one study, Sikora et al. (2012), looking at the ADHD symptoms in children with ASD, aged between 2 and 17.9years, divided the participants into groups based on whether their parents rated them as having clinically significant scores on ADHD problems subscales from the CBCL. The authors showed that those with ASD + ADHD symptoms had lower scores in several symptom scales: psychosocial health summary, school functioning, physical functioning, and emotional and social functioning scores were all lower than those of the children with ASD alone (Sikora et al., 2012).

In a more recent study, Lai et al. (2019) included 96 studies in a meta-analysis with the aim to evaluate the heterogeneity of ASD samples in terms of associated comorbidity diagnosis and symptoms. The authors found a pooled prevalence of ADHD comorbidity in ASD population of the 28%. Individuals with co-occurring ADHD and ASD are reported to show a range of other associated psychiatric and behavioral problems. The cumulative effects of the two disorders seem to lead to more severe impairments (Simonoff et al., 2008; Banaschewski et al., 2011; Lai et al., 2014) and poorer health-related quality of life (Lai et al., 2019) than those having ASD or ADHD alone.

An increased interest has been shown in the overlapping features between these disorders, including adaptive behaviors in children with ASD and ADHD co-occurrence. Mattard-Labrecque et al. (2013) investigated adaptive behaviors in children with overlap between ASD and ADHD compared to children with ADHD or ASD alone. The authors found that children with ASD and ADHD co-occurrence had lower adaptive behavior levels in all domains than children with ADHD or ASD alone, except in home/school domains, than children with ADHD (Mattard-Labrecque et al., 2013). In another study, no statistically significant differences emerged in adaptive functions between the ASD and ADHD group compared to the ASD group alone. However, the ASD and ADHD co-occurring phenotype shares inattention and hyperactivity deficit symptoms as well as emotional and behavior problems with the ADHD phenotype (Craig et al., 2015).

Moreover, according to the DSM-5 (American Psychiatric Association [APA], 2013), additional neurodevelopmental, mental, or behavioral conditions should be specified both in ADHD and in ASD, raising the need for a behavioral, emotional, and psychiatric evaluation. Furthermore, the elevated rate of medical disorders in children and adolescents with ASD and/or ADHD is associated with higher somatic problems (Muskens et al., 2017). The evaluation of psychological and medical conditions during the diagnostic assessment of children with ADHD, ASD, or the co-occurring phenotype, and follow-up examinations are then recommended to help determine risk factors and the most appropriate treatment.

However, although a large number of studies showed a high rate of ADHD and ASD combined diagnosis (Simonoff et al., 2008; Matson et al., 2013), few authors (McClain et al., 2017; Sokolova et al., 2017) have characterized the clinical features of a group with the co-occurrence diagnosis compared to ADHD or ASD groups separately in terms of associated psychopathological and behavioral profile. Sokolova et al. (2017), using a statistical causal model in a population of children with ASD and/or ADHD, found a significant and positive association between both inattention and impulsivity symptoms and difficulties in understanding social information, between hyperactivity symptoms and stereotypic and repetitive behaviors, and between both inattention symptoms and difficulties with understanding social information and verbal intelligence quotient (IQ). Other authors have confirmed that poor social skills in females with ADHD are comparable to those in children with ASD (Ohan and Johnston, 2011).

McClain et al. (2017) found that children with ASD, ADHD, and co-occurring ASD/ADHD exhibit similar inattention and hyperactivity/impulsivity levels conversely to previous findings that demonstrated that a dual diagnosis of ASD/ADHD is associated with more severe ADHD symptoms (Jang et al., 2013).

The Child Behavior Checklist (CBCL; Achenbach and Rescorla, 2001) is a well-established and widely used parent-completed measure of emotional and behavioral symptoms in children and adolescents aged 1.5–18 years (Sokolova et al., 2017; Guerrera et al., 2019). The CBCL results in a guided description of the child by the parents, whose fidelity in reporting symptoms is also widely recognized for psychopathological conditions and behavioral problems associated with ADHD and ASD, as recently shown by Guerrera et al. (2019).

To the best of our knowledge, no study has focused on studying the differences between the ADHD, ASD, and co-occurrence ADHD–ASD groups in terms of psychopathological and behavioral-associated symptoms using the CBCL scales. Furthermore, we found few previous studies that analyzed the differences between groups of children with ASD or ADHD only compared to those with the co-occurrence of ADHD and ASD. Finally, we have also found some inconsistencies in the results of these previous studies.

The main aim of this study was to try to better characterize a psychopathological and behavioral profile of the co-occurring phenotype of ADHD and ASD.

## Materials and Methods

The Child and Adolescent Neuropsychiatry Unit of Bambino Gesù Children’s Hospital, under the direction of the last author, upholds a comprehensive database made of several hundred patients. This database includes a wide range of information: anamnesis, family history, results from genetic analyses where available, information about past and current treatments (pharmacological and psychological treatments, speech therapy, etc…) and results from psychological and neuropsychological comprehensive evaluations, performed according to the good clinical practice recommended by international guidelines for neurodevelopmental disorder assessment.

Consistently with the aim of the current project, the patients who met our established inclusion criteria (described below) were retrospectively selected from this database. Our study is a retrospective observational study, and our institutional Ethic Committee has been notified according to the AIFA National Guidelines for Observational Study, in which retrospective studies do not require formal approval by the Ethics Committee.

Patients’ confidentiality was protected.

All procedures performed in studies involving human participants were in accordance with the ethical standards of the institutional and/or national research committee and with the 1964 Declaration of Helsinki and its later amendments or comparable ethical standards. Written informed consent was obtained from parents or legal guardians of each participant included in the study.

### Participants

The inclusion criteria comprised: the age between 6.0 and 16.11 (included); a diagnosis of ASD, ADHD, ASD + ADHD (primary diagnosis of ASD), or ADHD + ASD (primary diagnosis of ADHD); available results from a psychological evaluation, including a measure of cognitive level and at least one “golden standard” to support clinical diagnosis, the Autism Diagnostic Observation Schedule 2 (ADOS-2) for ASD and Conners’ Parent Rating Scale: Long Edition (CPRS) for ADHD diagnoses. Patients with suspected or ascertained genetic syndrome were excluded from the study.

In the period between September 2018 and June 2019, the Child and Adolescent Neuropsychiatry Unit of Bambino Gesù Children Hospital collected high-quality phenotype data of children who received a diagnosis of ADHD and/or ASD from two large datasets of 250 youths with ADHD and 250 youths with ASD (6–18 years of age). We included in the study 82 IQ- and age-matched individuals: 26 children with ADHD diagnosis, 30 with ASD diagnosis, and 26 with ADHD–ASD co-occurrence.

All the participants included in the database were previously assessed by an experienced multidisciplinary team, who performed an investigation of medical and developmental histories, as well as behavioral and diagnostic evaluations. The diagnoses of ASD and ADHD were based on the fifth version of the DSM; in addition to clinical assessment, ASD diagnoses were supported by the ADOS-2 (Lord et al., 2012). ADHD diagnosis was based on developmental history and extensive clinical examination and further supported by the evaluation of ADHD-related behaviors through the CPRS (Conners, 1997).

### Measures

#### Cognitive Measures

Cognitive development was preferably assessed by Wechsler Intelligence Scale for Children (WISC-IV; Wechsler, 2003). WISC-IV administration provides four different indexes: Verbal Comprehension Index (subtests: similarities, vocabulary, and comprehension); Perceptual Reasoning Index (subtests: block design, picture concepts, and matrix reasoning); Working Memory Index (subtests: digit span and letter–number sequencing); and Processing Speed Index (subtests: coding and symbol search).

In cases of failures in the completion of the WISC-IV for inadequacy of the language, mainly children with ASD, or for lack in attention, mainly children with ADHD, we administered Leiter-3 (Roid et al., 2013) or Colored Progressive Matrices (CPM; Raven et al., 1984), respectively.

The Leiter-3 offers a non-verbal measure of intelligence and evaluates the ability to reason by analogy and by matching and perceptual reasoning in general, irrespective of language and formal schooling. The non-verbal IQ obtained from the Leiter-3 is based on four subtests: Figure Ground, Form Completion, Classification and Analogies, and Sequential Order. CPM is a non-verbal assessment of intelligence. CPM is made up of 36 items appearing as a matrix reasoning test with a piece missing, which reduces the necessity for task instructions, for culture- or experience-dependent abilities, and for other specific abilities as fine motor or speech skills. The individual is asked to identify the correct response that completes the pattern, choosing from six alternative possible response options.

The Griffiths III (Green et al., 2016) was administered in only a few cases, when the child failed to complete the other cognitive scales because of his/her reduced attentional resources. The developmental quotient (DQ) obtained from Griffiths III is based on five subscales: Language and Communication Subscale, Eye and Hand Coordination Subscale, Personal, Social Emotional Subscale, and Gross Motor Subscale. Assessing proficiency in the activities of daily living, level of independence, and interaction with other children were employed as outcome measures.

ADOS-2 (Lord et al., 2012) is a semi-structured assessment tool allowing a systematic and standardized evaluation of the presence of ASD symptoms. It is considered a “gold standard” for collecting standardized and objective information about social communication skills, restricted interests, and repetitive behaviors, although it is insufficient on its own for a diagnosis. ADOS-2 comprises five modules: the Toddler Module for children aged 12–30 months without phrase speech, Module 1 for children aged 31 months and older without phrase speech, Module 2 for children with phrase speech but not verbally fluent, Module 3 for children and young adolescents with fluent language, and Module 4 for older adolescents and adults with fluent language.

Conners’ Parent Rating Scales-Long Version, Revised (Conners, 1997), are broadly used instruments for diagnostic and research purposes in the ADHD field, which can be administered to both parents and teachers. They assess core symptoms as well as symptoms of other behavioral and emotional disorders commonly associated with ADHD (e.g., oppositional behavior) based on DSM-IV-TR (American Psychiatric Association [APA], 1994) criteria (Sparrow, 2010).

The CBCL, Ages 6–18 (Achenbach and Rescorla, 2001) was used to assess comorbid psychiatric symptomatology using parents’ ratings. CBCL items investigate emotional and behavioral problems over the previous 6 months, with three response options (0 = not true, 1 = somewhat or sometimes true, 2 = very true or often true).

The CBCL 6–18 questionnaire consists of two parts: one addressing social competence and the other for assessing emotional and behavioral problems in children aged 4–18 years. In the study, only the latter part was used. The questionnaire includes a 118-item scale yielding several subscales, including syndrome scales (Withdrawn, Somatic Complaints, Anxious/Depressed, Social Problems, Thought Problems, Attention Problems, Delinquent Behavior and Aggressive Behavior, a Total Problem Score) and two broadband scores, Internalizing Problems and Externalizing Problems.

The Internalizing domain incorporates three syndrome scales: Anxious/Depressed, Withdrawn/Depressed, and Somatic Complaints. The Externalizing domain incorporates the Rule-Breaking Behavior and Aggressive Behavior syndrome scales. The Total Problems scale is based on responses to all CBCL items, including Social Problems, Thought Problems, and Attention Problems scales. DSM-oriented scales included affective, somatic, and anxiety problems; ADHD; oppositional/defiant problems; and conduct problem. CBCL also includes three additional scales, the 2007 scales, namely, Sluggish Cognitive Tempo, Obsessive-Compulsive, and Post-Traumatic Stress Disorder scales.

All scales have a t-score mean of 50 and a standard deviation of 10, and different norms are provided for gender across age groups. According to the normative data of the CBCL, a *t*-score ≤64 indicates non-clinical symptoms, a *t*-score between 65 and 69 indicates problems rated high enough to be of concern but not overtly deviant, and a *t*-score ≥70 indicates clinical symptoms. For the subscales “internalizing,” “externalizing,” and “total” problems, a *t*-score ≤59 indicates non-clinical symptoms, a t-score between 60 and 64 indicates that the child is at risk for problem behaviors, and a *t*-score ≥65 indicates clinical symptoms.

### Statistical Methods

#### Demographic Variables

Analyses of variances (ANOVAs) were used for group comparisons between the ASD, the ADHD, and the ASD + ADHD groups (age and IQ). The chi-squared test was performed on categorical variables. ADOS-2 comparative scores of ASD and comorbidity groups, as well as CPRS scores of ADHD and comorbidity groups, were compared by means of *t*-tests.

The following analyses were conducted: descriptive statistics (mean and standard deviations; percentage of non-clinical, borderline, and clinical scores) for 17 CBCL subscales separated for diagnostic group (ASD, ADHD, co-occurrence ADHD and ASD); multivariate analysis of variance (MANOVA) to evaluate the impact of diagnosis on CBCL subscales’ scores, with group (ASD, ADHD, comorbidity) as a between-subject factor and 17 CBCL subscale T-scores as within-subject factors: anxious/depressed, withdrawn/depressed, somatic complaints, social problems, thought problems, attention problems, rule-breaking behavior, aggressive behavior, internalizing problems, externalizing problems, total problems, affective problems, anxiety problems, somatic problems, ADHD, oppositional/defiant, conduct problems; Bonferroni *post hoc* analyses were conducted; and comparisons across all groups were made. Finally, for the examination of the relationship between age, sex, and T-scores on internalizing and externalizing problems in CBCL subscales, additional Pearson and Spearman correlations were applied.

All statistical tests were based on a significance level of *p* < 0.05.

Statistical analyses were performed using the Statistical Package for the Social Sciences, version 13.0 (IBM Corp., Armonk, NY, United States).

## Results

### Demographic and Clinical Features

Demographic features of the sample are summarized in Table 1. As concerns the cognitive measures used for the assessment of the ADHD group, 14 out of 26 youths (54%) were evaluated by means of the WISC-IV, seven (27%) by means of CPM, and the remaining five (19%) by means of Leiter-3, whereas 23 out of 30 youths (77%) belonging to the ASD groups were evaluated by means of Leiter-3, five (17%) were evaluated by means of WISC-IV, and only two children were evaluated through CPM and Griffiths III. Finally, as concerns the ADHD and ASD co-occurrence group, 13 out of 26 youths (50%) were evaluated by means of Leiter-3, eight (31%) were evaluated through WISC-IV, three (11.5%) were assessed through CPM, and two (7%) were assessed by Griffith III.

**TABLE 1:** Demographic characteristics and cognitive and psychopathological measures of children and adolescents included.

	ADHD (*n* = 26)	ASD (*n* = 30)	Co-occurrence ADHD-ASD (*n* = 26)	*p-*values
Age (mean and SD)	9.6 (3)	9.1 (2.6)	9.5 (3.1)	0.800
Sex (M/F)	19/7	24/6	22/4	0.586
IQ or DQ (mean and SD)	91.5 (19.5)	82.3 (20.6)	82.8 (22.1)	0.194
ADOS-2 (mean and SD)	–	6.8 (0.9)	6.2 (1.5)	0.088
CPRS – ADHD scale (mean and SD)	84 (6.4)	–	73.6 (11.9)	0.0003

*ADHD, attention deficit/hyperactivity disorder; ASD, autism spectrum disorder; co-occurrence ADHD–ASD; DQ, developmental quotient; IQ, intelligence quotient; ADOS-2, Autism Diagnostic Observation Schedule-2 Comparative Score; CPRS, Conners’ Parent Rating Scales-ADHD scale. ANOVAs indicated that the cohorts had similar population characteristics regarding age at evaluation, sex, and IQ (*p* > 0.05). ASD and co-occurrence ADHD–ASD groups did not significantly differ on ADOS-2 comparative scores (*p* > 0.05). ADHD scale of CPRS was used to compare ADHD severity in ADHD and co-occurrence ADHD–ASD groups: a statistically significant difference emerged (*p* < 0.001).*

### Psychopathological Profile: Child Behavior Checklist Scores Across Diagnoses

**FIGURE 1:**
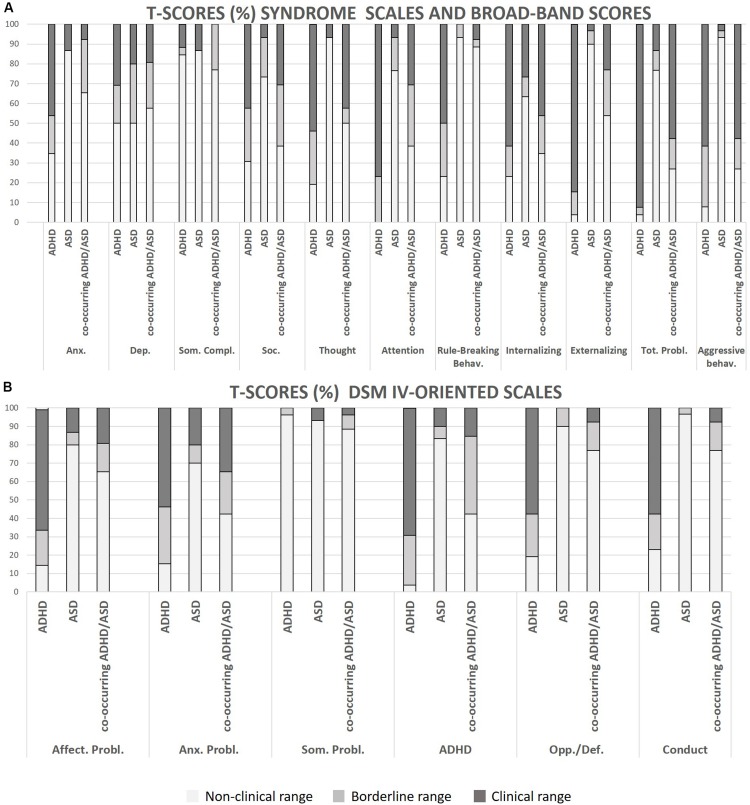
**(A,B)** Representations of the distributions of CBCL scores (clinical, borderline and non-clinical). *Anx.*, Anxious/depressed; *Dep.*, withdrawn/depression; *Soc.*, social problems; *Thought*, thought problems; *Tot. Probl*., Total Problems; *Affect. Probl.*, affective problems; *Anx. Probl*., anxiety problems; *Som. Probl.*, somatic problems; *Opp./Def.*, oppositional/defiant; *Conduct*, conduct problems.

Qualitative representations of the distributions of CBCL in clinical, borderline, and non-clinical scores for each group are provided in Figures 1A,B. Means and standard deviations of the scores in the selected CBCL subscales were calculated for each group (Table 2).

**TABLE 2:** Group differences on CBCL scales (means and standard deviations).

	ADHD	ASD	Co-occurrence ADHD–ASD	*p*-values
Anxious/Depressed	66.9 (8.1)	56.3 (8.3)	59.8 (7.5)	<0.000
Withdrawn/Depressed	65.1 (9.8)	61.7 (8.3)	63.1 (10.4)	0.416
Somatic Complaints	58.8 (6.3)	55.1 (7.9)	56.5 (7.1)	0.151
Social Problems	69.5 (9.8)	60.3 (5.9)	64.7 (7.9)	<0.000
Thought Problems	68.6 (8.1)	59.1 (7.9)	66.3 (7.7)	<0.000
Attention Problems	74.5 (6)	61.2 (10.11)	68.3 (8.4)	<0.000
Rule-Breaking Behavior	68 (8.1)	54.5 (4.8)	56 (11.6)	<0.000
Aggressive Behavior	75.9 (10.7)	53.9 (5.2)	59.1 (6.7)	<0.000
Internalizing Problems	65.6 (7.3)	56.4 (10.2)	60.1 (9.3)	0.002
Externalizing Problems	71.8 (8)	51.2 (7.7)	59.1 (6.6)	<0.000
Total Problems	72.7 (5.2)	56.5 (7.9)	63.9 (7.4)	<0.000
Affective problems	70.4 (6.9)	57.7 (9.4)	61.8 (7.5)	<0.000
Anxiety Problems	68.6 (7.1)	59.8 (8.2)	64.6 (7.6)	<0.000
Somatic problems	56.1 (5.4)	54.2 (7.9)	55 (6.9)	0.607
ADHD Problems	72.4 (5.1)	57.5 (7)	64.5 (6.6)	<0.000
Oppositional/Defiant Problems	69.2 (7.5)	53.8 (4.9)	57 (7.1)	<0.000
Conduct Problems	69.4 (9.1)	53.1 (4.1)	58.3 (6.5)	<0.000

*The comparison of the ADHD, ASD and co-occurrence ADHD–ASD samples applying a MANOVA with group as the between-subject factor, revealed significant group differences for almost all scores of the considered CBCL scales. Results are summarized in Table 2.*

Bonferroni *post hoc* analyses were conducted; comparisons across all groups were made. As expected, comparisons between ADHD and ASD groups were statistically significant (*p* ≤ 0.001) for all the considered CBCL scales. Depression, Somatic Problems and Somatic Complaints scales did not significantly differ between the two groups (*p* > 0.05). Comparisons between the ADHD and the co-occurrence ADHD-ASD groups revealed statistically significant differences in the following scales: Anxiety (*p* = 0.006), Attention (*p* = 0.029), Rule-Breaking Behavior (*p* < 0.000), Aggressive Behavior (*p* < 0.000), Externalising Problems (*p* < 0.000), Total Problems (*p* < 0.000), Affective Problems (*p* = 0.001), ADHD (*p* < 0.000), Oppositional/Defiant (*p* < 0.000), and Conduct Problems (*p* < 0.000). Comparisons between co-occurrence ADHD–ASD and ASD groups revealed statistically significant differences in the following scales: Thought Problems (*p* = 0.003), Attention (*p* = 0.008), Aggressive Behavior (*p* = 0.047), Externalising Problems (*p* < 0.000), Total Problems (*p* = 0.001), ADHD (*p* < 0.000), and Conduct Problems (*p* = 0.016).

## Discussion

The main aim of the present study was to characterize a psychopathological and behavioral profile of the co-occurring ADHD–ASD phenotype.

We analyzed three subgroups of a large dataset of outpatients diagnosed for ADHD, ASD, and the ADHD–ASD co-occurrence groups at the Child and Adolescents Psychiatric Unit at the Bambino Gesù Children’s Hospital in Rome.

The new DSM-5 (American Psychiatric Association [APA], 2013), allowing for a dual diagnosis, has contributed to the increasing interest of clinical researchers studying the comorbid phenotype. We learned by several previously reported authors (Ronald et al., 2008; Simonoff et al., 2008; Banaschewski et al., 2011; Lai et al., 2014; Lecavalier et al., 2019) that ADHD and ASD frequently co-occur.

Our results showed that in the co-occurrence ADHD–ASD phenotype, the externalizing dimensions, obtained from the CBCL Externalizing Problems scales, as well as Thought Problems, Attention scores, Aggressive Behaviors, ADHD scores, and Conduct Problems are higher than those in the ASD group but lower than those in the ADHD group. Our preliminary findings revealed that the externalizing symptom scales of the CBCL scores significantly differed between the co-occurring ADHD–ASD group and the other two groups. This finding supports the hypothesis of the existence of an intermediate and independent phenotype between ADHD and ASD, which seems to be defined by the externalizing problems dimension.

Furthermore, we found that a representative prevalence of the internalizing problems, as well as Depression, Somatic Complaints, and Somatic Problems scales obtained from the CBCL, does not significantly differ between the co-occurring ADHD–ASD phenotype and the other two groups. This finding means that internalizing problems may overlap across the three groups.

Our findings also confirm the high sensitivity of the CBCL for the internalizing symptoms previously described around 30%, in line with Guerrera et al. (2019). This result confirms that CBCL is a specific instrument to measure internalizing symptoms in ASD.

Our study has some limitations. First, our analyses examined a limited number of children and adolescents with the co-occurring ADHD–ASD phenotype. Therefore, we look at our results as preliminary findings, and we are working to expand the size of each group. Furthermore, in this study, we evaluated the differences across groups. Thus, future studies using dimensional analyses focused on internalizing and externalizing symptom dimensions and in groups with a bigger sample size are needed. Moreover, this is a retrospective study using diagnostic tools according to parental judgment. So, future studies will have perspective designed with a consistent long-term follow-up. Finally, parents alone completed CBCL, while collecting reports from both parents and teachers may be more informative. Also, in the current study, we did not have any report about the adaptive behaviors nor interviews led by clinicians [i.e., Kiddie Schedule for Affective Disorders and Schizophrenia (K-SADS)] which may explain what symptom associated with ASD and ADHD is the most disabling. In the next studies, reports from teachers and interviews led by clinicians will be included to integrate parent’s reports and as a measure of the adaptive behaviors.

## Conclusion

In summary, results suggested that CBCL has been confirmed to be a very accurate instrument to detect the psychopathological, symptoms, and emotional and behavioral profile of the associated comorbidities in children and adolescents with ASD, ADHD, and the co-occurrence condition.

Our preliminary findings suggested the existence of an intermediate and independent phenotype between ADHD and ASD that seems to be defined through the externalizing symptom dimensions.

Further studies are needed to analyze the overlap of symptoms in the three groups with dimensional analyses and in order to reinforce the current results.

## Data Availability Statement

The datasets generated for this study are available on request to the corresponding author.

## Ethics Statement

The studies involving human participants were reviewed and approved by The Ethics Committee of the IRCCS Bambino Gesù Children’s Hospital of Rome. Written informed consent to participate in this study was provided by the participants’ legal guardian/next of kin.

## Author Contributions

AC participated the design, coordination of the study and interpretation of the data, performed the measurement, drafted, and revised the manuscript. EF conceived the study, participated in its design and interpretation of the data, performed the statistical analysis, and helped to draft and revise the manuscript. SG, EN, and GV participated in the design of the study, performed the measurement, and helped to draft and revise the manuscript. SV participated in the design of the study and coordination, interpretation of the data, and helped to draft the manuscript. All authors read and approved the final manuscript.

## Conflict of Interest

The authors declare that the research was conducted in the absence of any commercial or financial relationships that could be construed as a potential conflict of interest.
